# Infiltrating Cardiac Synovial Sarcoma Presenting as Acute Cerebrovascular Accident

**DOI:** 10.1155/2017/8539606

**Published:** 2017-12-03

**Authors:** Kelechukwu U. Okoro, Matthew D. Roby, David C. Sane, Robert E. Budin

**Affiliations:** ^1^Department of Internal Medicine, Virginia Tech Carilion–School of Medicine and Research Institute, Roanoke, VA, USA; ^2^Department of Internal Medicine, Section of Cardiology, Virginia Tech Carilion–School of Medicine and Research Institute, Roanoke, VA, USA; ^3^Department of Pathology, Virginia Tech Carilion–School of Medicine and Research Institute, Roanoke, VA, USA

## Abstract

Primary cardiac sarcoma is a rare malignant myocardial neoplasm that does not exhibit gender predominance or age predilection. The classification of these tumors includes several subtypes, of which synovial sarcoma is a rare manifestation. When present, these tumors portend a poor prognosis with high morbidity and mortality that is attributable to their inherent infiltrative capacity, especially in the absence of treatment. The general consensus for treatment is surgical excision and neoadjuvant chemotherapy and radiotherapy. In this report, a case of synovial sarcoma involving the left ventricular outflow tract and aortic valve is presented.

## 1. Introduction

Cardiac tumors can be classified as either primary or secondary [1]. Secondary tumors are usually metastasis from other primary sites distal to the heart and are far more prevalent than primary tumors [1–3]. Primary cardiac tumors are classified into benign and malignant tumors, with benign cardiac tumors comprising approximately 75% of all primary cardiac tumors [2–4]. Although rare, the bulk of malignant cardiac tumors are sarcomas, most commonly angiosarcoma and rhabdomyosarcoma, but other forms of sarcoma may also be present at times [2–4]. These tumors do not exhibit gender predominance or age predilection, tend to grow in any location of the heart, and grow exponentially [3, 5, 6]. Distant metastasis may occur to sites such as liver, bone, and brain [6].

Cardiac synovial sarcoma (SS) is a unique form of sarcoma that is infrequently encountered. It makes up approximately 5% of cardiac sarcomas and less than 1% of all primary cardiac tumors [7]. It is usually found in the atria, right side of the heart, or pericardial surface; arises from mesenchymal stem cells; and comprises four subcategories [1, 5]. SS remains asymptomatic until it infiltrates vital structures, obstructs cardiac output, or embolizes [3]. When symptomatic, patients can experience dyspnea, orthopnea, pyrexia, dysrhythmia, and angina [5].

## 2. Case Presentation

A 41-year-old G2P1001 parous female whose past medical history is pertinent for remote migraines presented to our facility from an outside hospital (OSH) with complaints of sudden onset of left-sided hemiplegia, facial paralysis, and dysarthria. Her symptoms resolved spontaneously en route to the ED. The pregnancy had been uneventful except for a period approximately 6 weeks prior to presentation where she gained eight pounds in one week, prompting evaluation for preeclampsia. During that workup, she was found to have mildly elevated liver function tests (LFTs) and thrombocytopenia without hypertension or proteinuria. Serial ancillary evaluations revealed improved parameters; thus, no further inquests were made. Upon presentation to the OSH, an emergent CT head did not reveal any signs of acute disease. Labs revealed white blood cell count 8.3K/μl, hemoglobin 11.9 g/dl, hematocrit 35.2%, Plt 87K/μl, sodium 135 mmol/L, potassium 3.3 mmol/L, chloride 103 mmol/L, BUN 16 mg/dl, creatinine 0.63 mg/dl, AST 56 U/L, ALT 27 U/L, INR 1.55, D-dimer 13.48 mg/L, and troponin 0.24.

**Figure 1:**
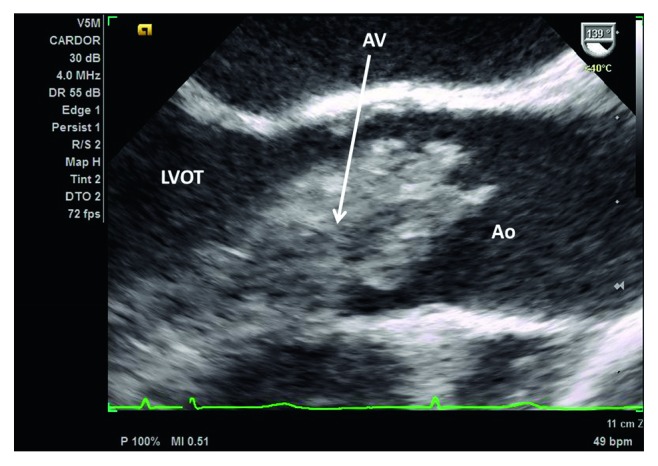
17 mm × 18 mm mobile obstructing mass within the left ventricular outflow tract attached to the aortic valve and the interventricular septum. LVOT = left ventricular outflow tract, AV = aortic valve, and Ao = aorta.

**Figure 2:**
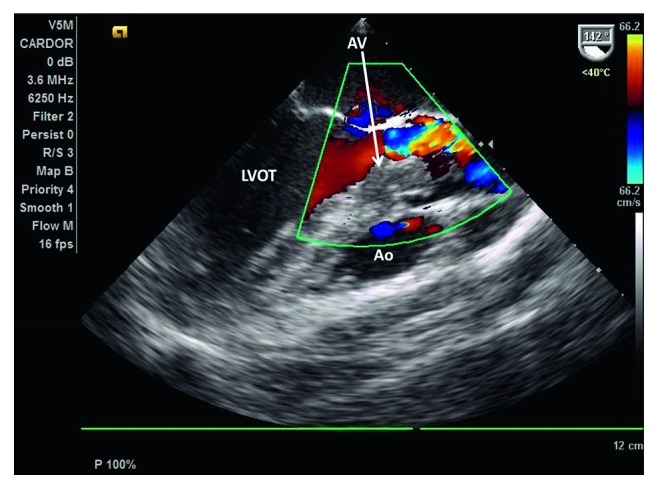
Doppler showing functional aortic stenosis and regurgitation.

**Figure 3:**
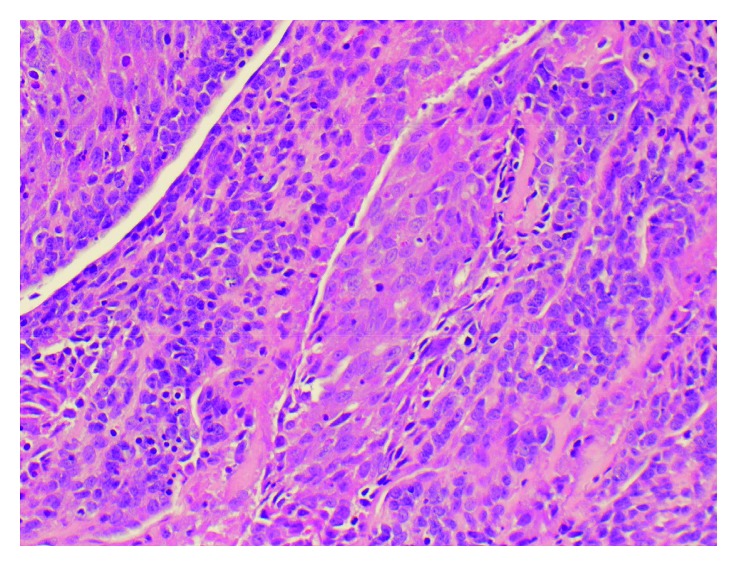
Biphasic synovial sarcoma with both spindle- and epitheliod-type features (H&E staining, 400x).

**Figure 4:**
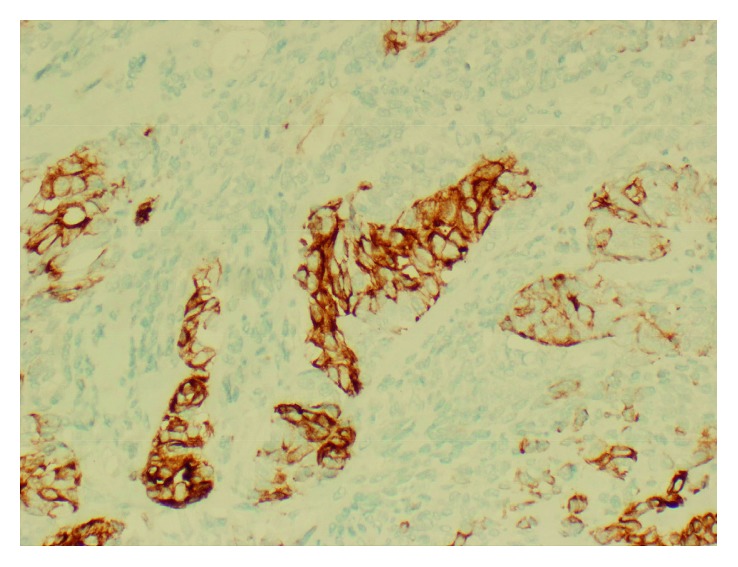
Keratin AE1/AE3 IHC demonstrating the presence of epithelioid components (200x).

**Figure 5:**
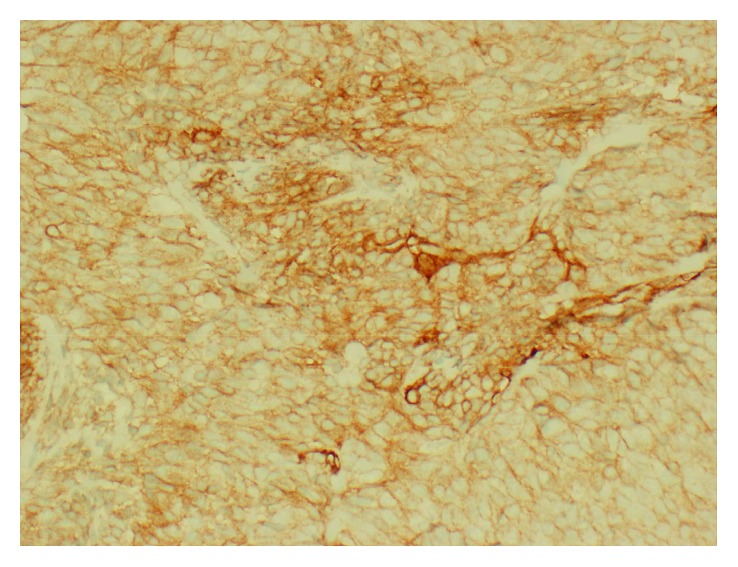
Positive CD99 IHC in both epithelioid and spindle cells (200x).

**Figure 6:**
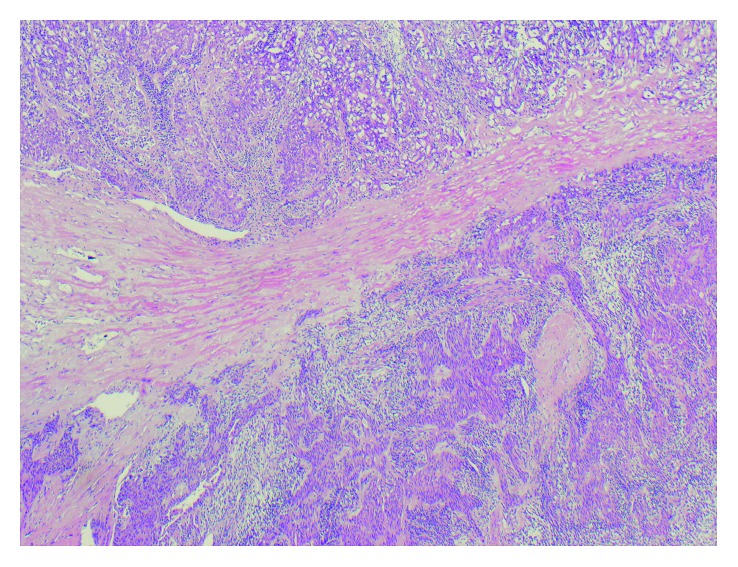
H&E staining showing cardiac myocyte invasion (40x).

**Figure 7:**
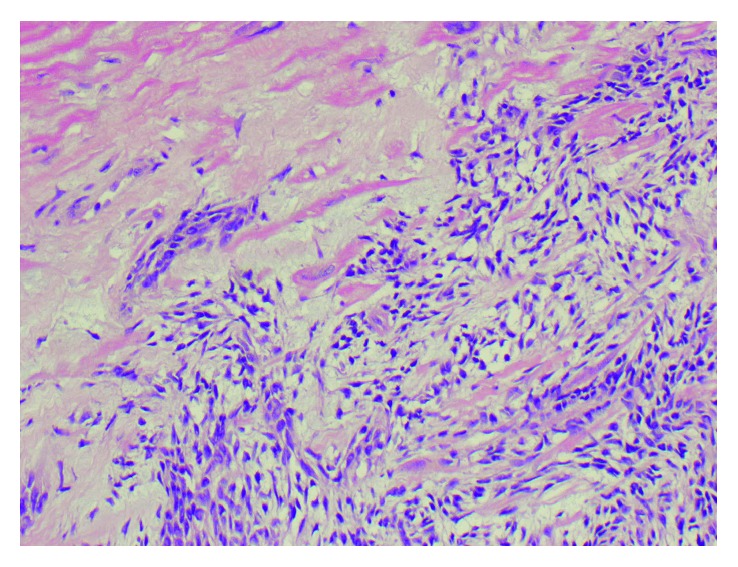
H&E staining showing cardiac myocyte invasion (200x).

**Figure 8:**
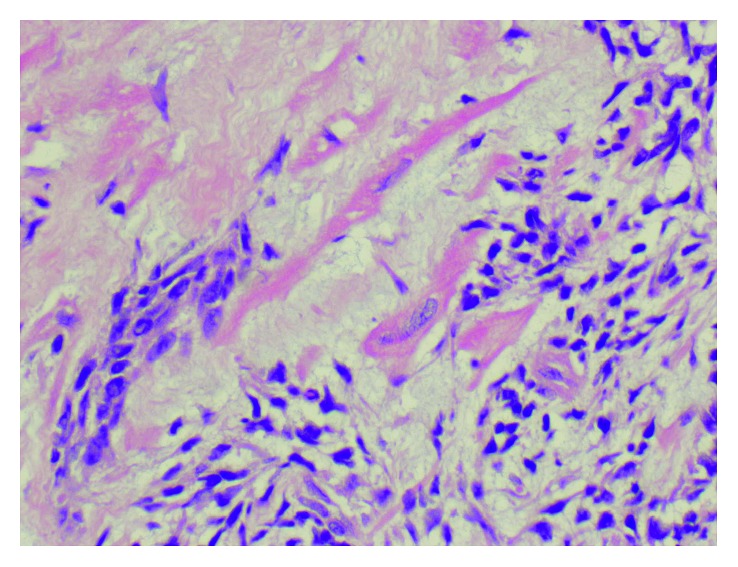
H&E staining showing cardiac myocyte invasion (400x).

Stat pelvic ultrasound was performed upon arrival to our facility which showed normal fetal heart and respirations. MRV/MRA demonstrated a 7 × 12 mm area of acute infarct in the right posterior insular cortex with normal cerebral arteries and venous sinuses. Transthoracic echocardiography (TTE) revealed preserved ejection fraction of 65–70% and a 17 × 18 mm obstructing mobile mass possibly attached to the aortic valve or the left ventricular outflow tract (LVOT) with an aortic valve area of 0.52 cm^2^ and peak and mean gradients of 57 mmHg and 33 mmHg, respectively. Follow-up TEE confirmed TTE findings (Figures 1 and 2). As a result, a shared decision was made to proceed with stat C-section at 35 w 2 d. The operation was successful yielding a healthy female infant. The next day, the patient returned to the OR for resection of the cardiac mass. Frozen section during the operation confirmed primary cardiac synovial sarcoma with histologic analysis of the cardiac mass revealing a biphasic synovial sarcoma (Figure 3) positive for cytokeratin and CD99 (Figures 4 and 5). The specimen also stained positive for calretinin and EMA. Cytogenetic testing demonstrated t(X;18)(p11.2;q11.2) gene translocation. The neoplasm involved the right coronary aortic valve leaflet and both muscular and membranous parts of the IV septum (Figures 6–8). As a result, the surgeon proceeded with the Bentall and modified Konno procedures after wide resection of the mass. The operation was a success, but unfortunately, the patient went into cardiorespiratory arrest on postoperative day one. She was resuscitated and placed on vasopressors and inotropes but remained pacer dependent. Unfortunately, despite aggressive treatment, the patient expired.

## 3. Discussion

The differential diagnosis for intracardiac masses includes tumors, thrombi, vegetation, and calcific lesions amongst others [1]. Based on the size, motion, and echogenicity of the mass, it was believed to be a tumor. As previously mentioned, cardiac masses can either be of primary cardiac origin or be secondary as a result of distant metastasis. The primary tumors comprise benign and malignant tumors of which sarcoma makes up the bulk of the malignant tumors. A study determined the prevalence of primary cardiac sarcoma at autopsy to be 0.00017%, with an incidence from 0.001 to 0.03% and a general incidence from 0.07% [5].

Based on histology, there are multiple types of cardiac sarcoma. One of the rare forms of cardiac sarcoma is synovial sarcoma. It has not been described extensively in literature due to its low incidence and prevalence. As the name implies, SS is of mesenchymal origin. It makes up approximately 5% of cardiac sarcomas and less than 0.1% of all primary cardiac tumors [7]. It comprises four subcategories and is commonly found in the atria, right side of the heart, or pericardial surface with left-sided occurrence a rarity [1, 5, 8, 9]. The subcategories of SS include biphasic, monophasic fibrous, monophasic epithelial, and poorly differentiated [10]. All four subtypes are characterized by the composition of the two cellular elements and the degree of differentiation. The two main cellular elements that can be seen on histology include epithelial cells and spindle cells [10, 11]. Immunohistochemical staining for epithelial and mesenchymal markers such as EMA, S-100, CD99, cytokeratin, and BCL2 (Table 1) can be used as an adjunct to histologic analysis to diagnose SS.

The hallmark for diagnosing synovial sarcoma is the cytogenetic detection via FISH of the translocated chromosome t(X;18)(p11.2;q11.2) which is present on approximately 90% of synovial sarcomas [9–12]. This translocation results in the fusion of the *SYT* gene on chromosome 18q11 with the *SSX1*, *SSX2*, or *SSX4* gene on chromosome Xp11 [10, 11, 13]. Normally, the SSX and SYT genes encode for proteins that function as transcription regulators in the cell nucleus. The SSX codes for a transcription inhibitor protein, while the SYT codes for a transcription activator protein. The translocation results in replacement of the inhibitor region of SSX with the activator portion of SYT. It is thought that the protein from the fusion SYT-SSX oncogene likely regulates chromatin remodeling which may lead to enhanced proliferation of mesenchymal cells [11]. The fusion oncogene has also been associated with aberrant E-cadherin repression, Bcl-2 overexpression, and downregulation of Mcl1 [13].

For all cardiac sarcomas, the definitive therapy is excision, which may prove difficult due to the intrinsic infiltrative nature of the tumors with concomitant administration of neoadjuvant chemotherapy and radiotherapy [3, 5, 13]. In our patient, the mass was attached to the muscular septum, membranous septum, and the right coronary leaflet. This prompted a shared discussion between the patient, her family, and the medical team culminating in the agreement to proceed with a wide resection of the mass by removing the muscular septum, the membranous septum, and dissecting into the right ventricular outflow tract (RVOT) as a modified Konno procedure. After successful resection, a modified Bentall procedure was used to replace the aortic root along with the aortic valve via a porcine bioprosthesis, reimplant the coronary vessels, reconstruct the interventricular septum, the RVOT, and repair the tricuspid valve.

The data on neoadjuvant chemotherapy are limited due to the rarity of the disease and absence of double-blinded controlled studies. The general consensus regarding chemotherapy is administration of a combination of an anthracycline such as daunorubicin or doxorubicin and a nitrogen mustard such as cyclophosphamide or ifosfamide with the adriamycin-ifosfamide combination showing the most consistent efficacy. Despite excision and cytotoxic therapy, the likelihood of recurrence is high with several manuscripts reporting recurrence after therapy [8, 9]. Molecular targeting seems to be a great avenue for further research with the prevalence of t(X;18) translocation in SS. Several studies have shown that knockdown of the SYT-SSX oncogene results in decreased cell viability secondary to an increase in apoptosis [13]. Without treatment, prognosis is usually grim with predicted 6–24 month survival even in the presence of adjuvant therapy [5, 6, 10]. The main prognostic factor is complete resection of the tumor [3, 5, 10]. There have been reports of improved recurrence rate and overall survival rate with adjuvant chemotherapy [5, 10].

## 4. Conclusion

Synovial sarcoma is a rare form of cardiac sarcoma that portends a dismal prognosis. Further studies are required to determine appropriate treatment strategies.

## Figures and Tables

**Table 1:** Immunohistochemical panel of antibodies utilized in determining diagnosis of synovial sarcoma.

Immunohistochemistry	Results
EMA	+
Calretinin	+
CD99	+
Cytokeratin	+
CD34	−
CD31	−
Desmin	−
Synaptophysin	−
S-100	−
Melan A	−
CD45	−
Vimentin	+
Thyroid transcription factor	−
